# Supplementary figures and images for: Urine Flow Cytometry and Dipstick Analysis in Diagnosing Bacteriuria and Urinary Tract Infections among Adults in the Emergency Department—A Diagnostic Accuracy Trial
Source: Diagnostics (Basel). 2024 Feb 13;14(4):412. doi: 10.3390/diagnostics14040412 (PMC10888022; doi:10.3390/diagnostics14040412)

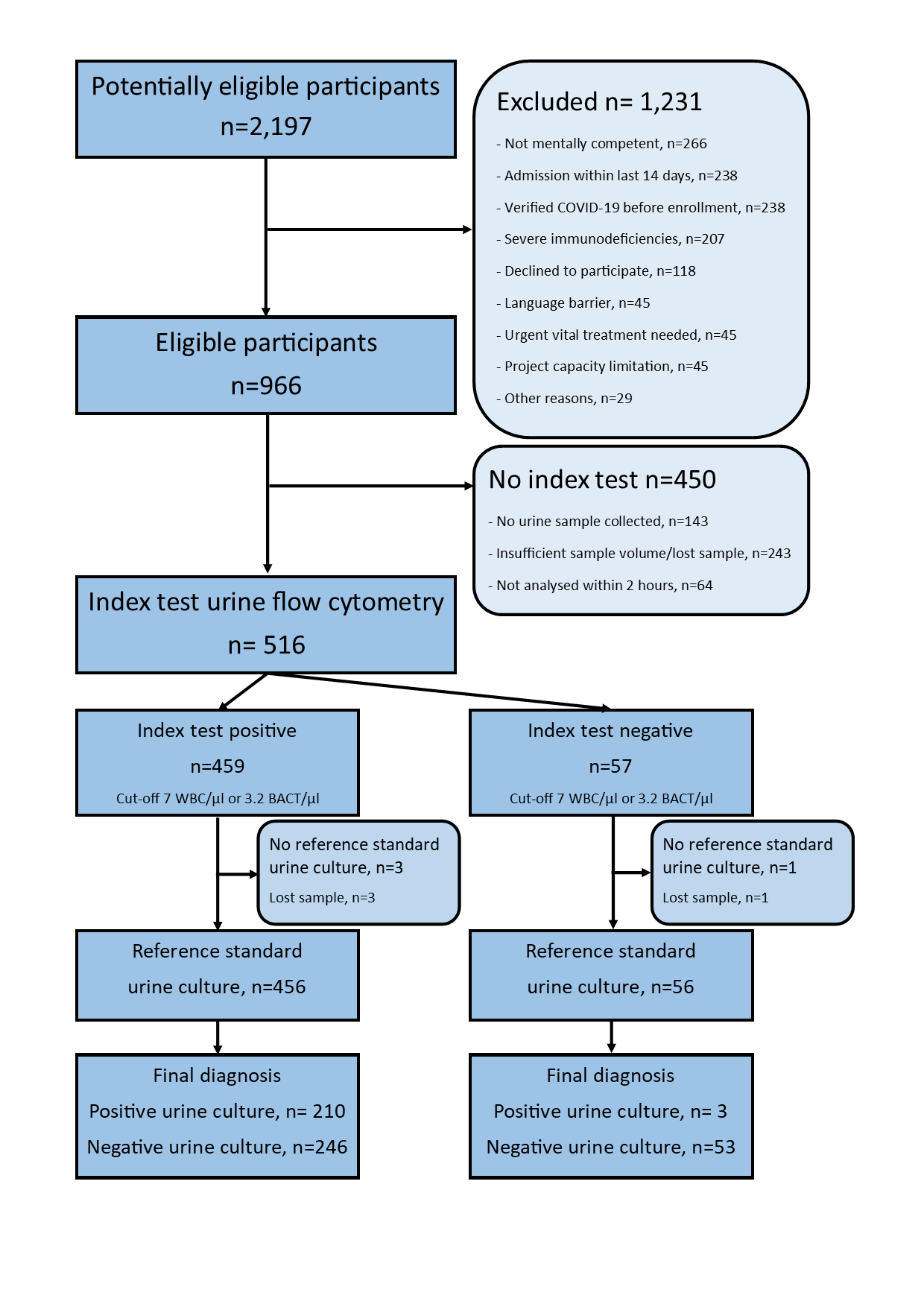

Supplement: Supplementary file 1 [file diagnostics-14-00412-s001.zip › Figure S1 STARD flowchart index test UFC reference test bacteriuria model WBC or BACT.png]

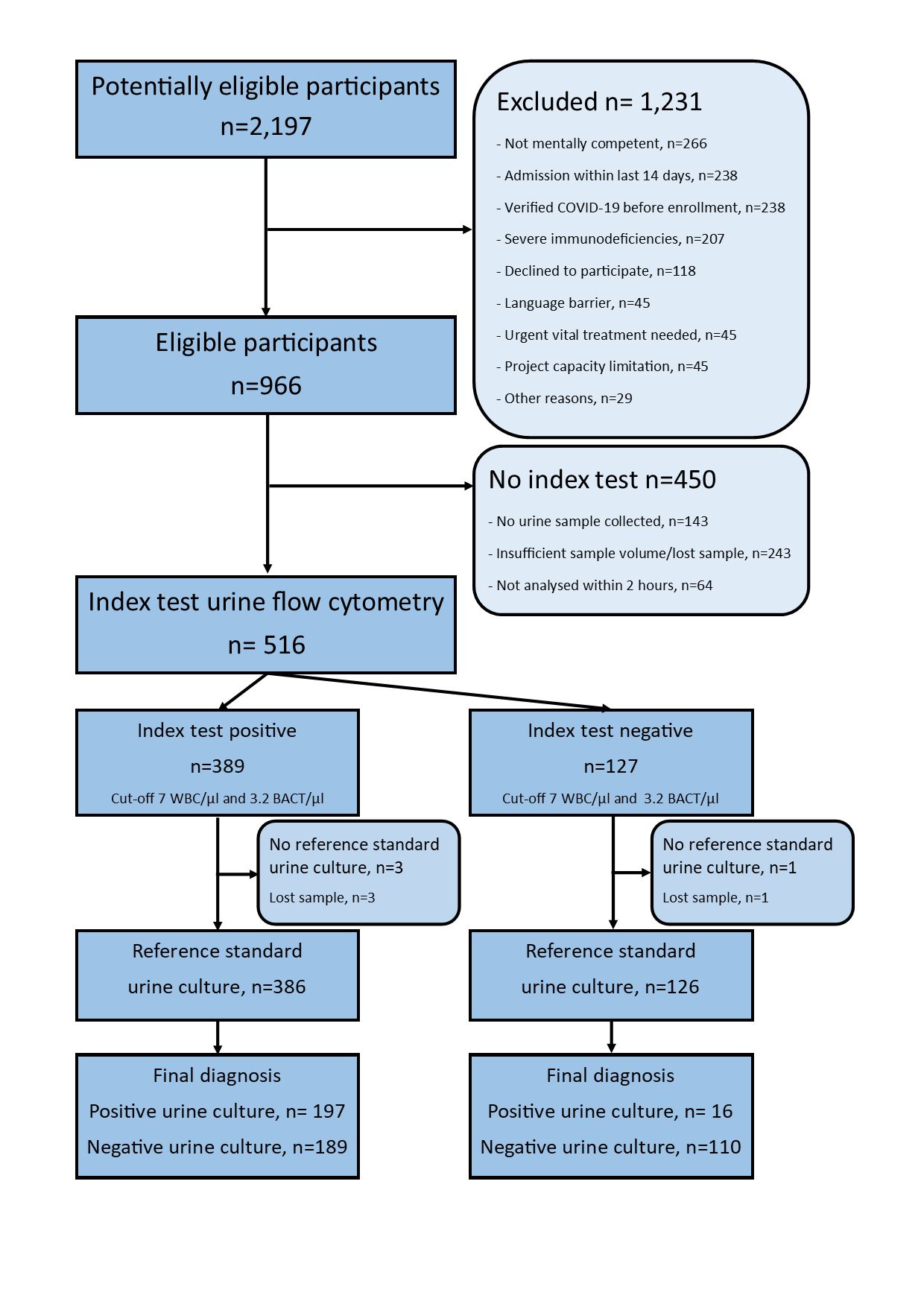

Supplement: Supplementary file 1 [file diagnostics-14-00412-s001.zip › Figure S2 STARD flowchart index test UFC reference test bacteriuria model WBC and BACT.png]

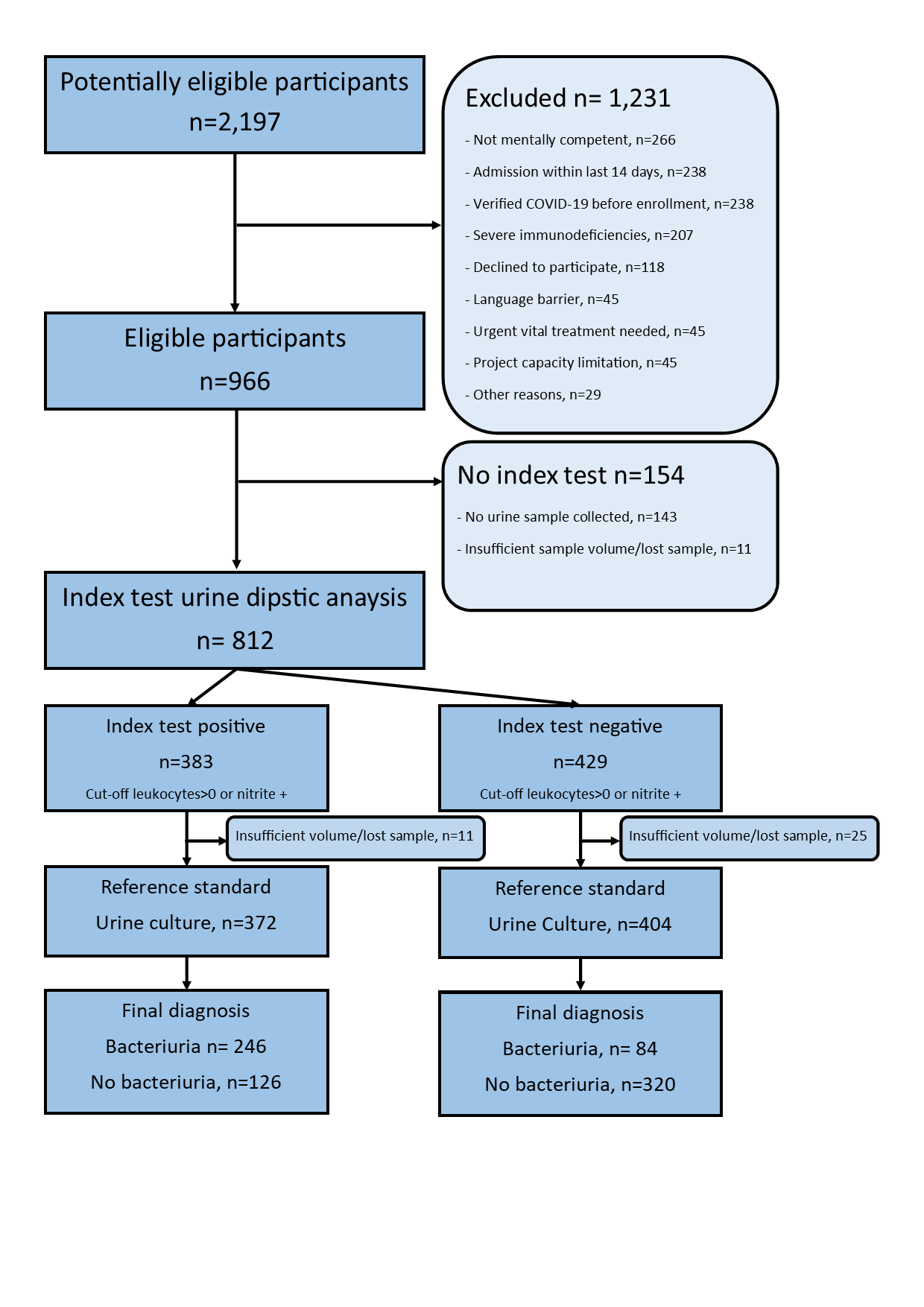

Supplement: Supplementary file 1 [file diagnostics-14-00412-s001.zip › Figure S3 STARD flowchart index test UDA reference test bacteriuria model LKC+ or Nitrite+.png]

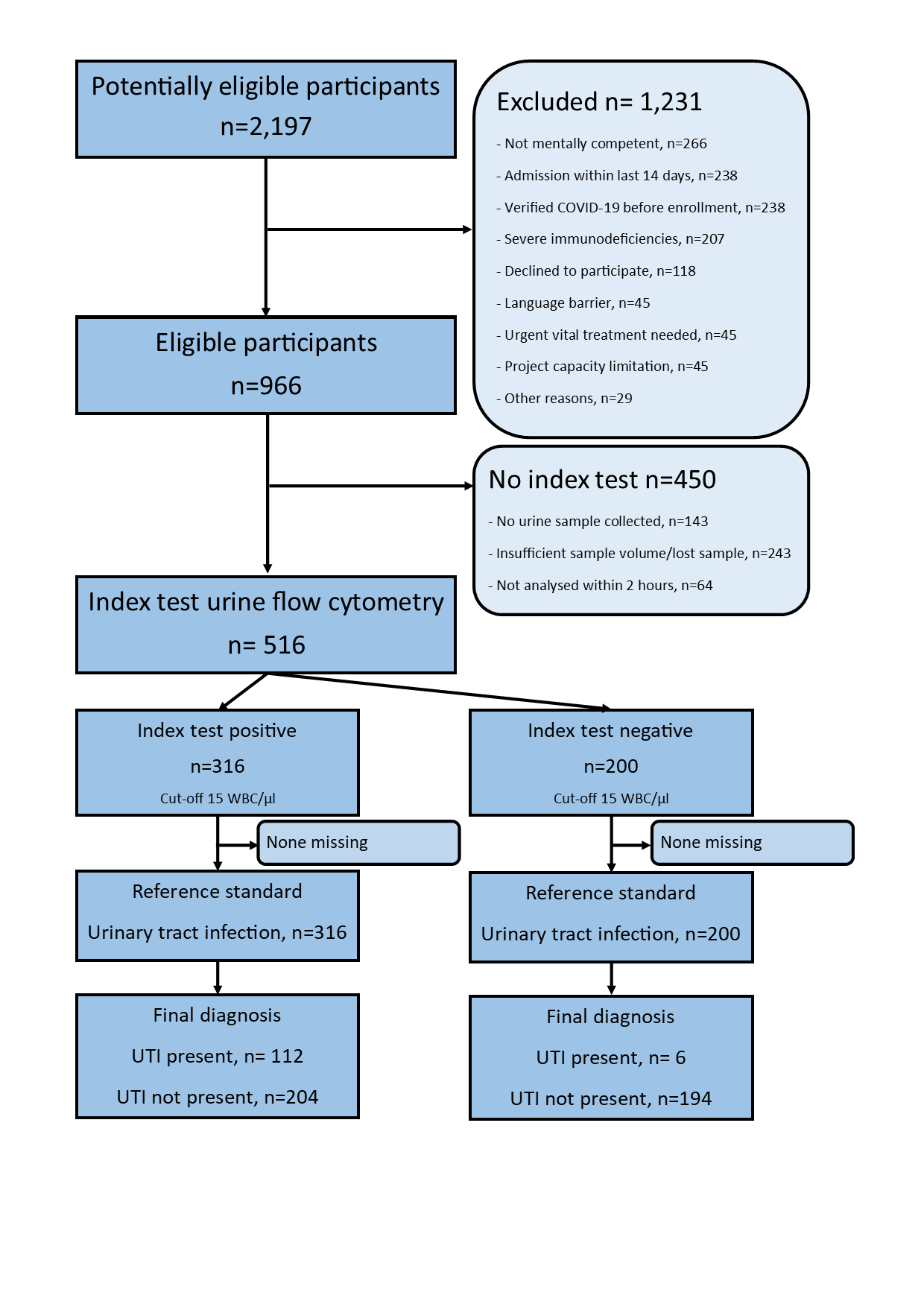

Supplement: Supplementary file 1 [file diagnostics-14-00412-s001.zip › Figure S4 STARD flowchart index test UFC reference test UTI model WBC15.png]

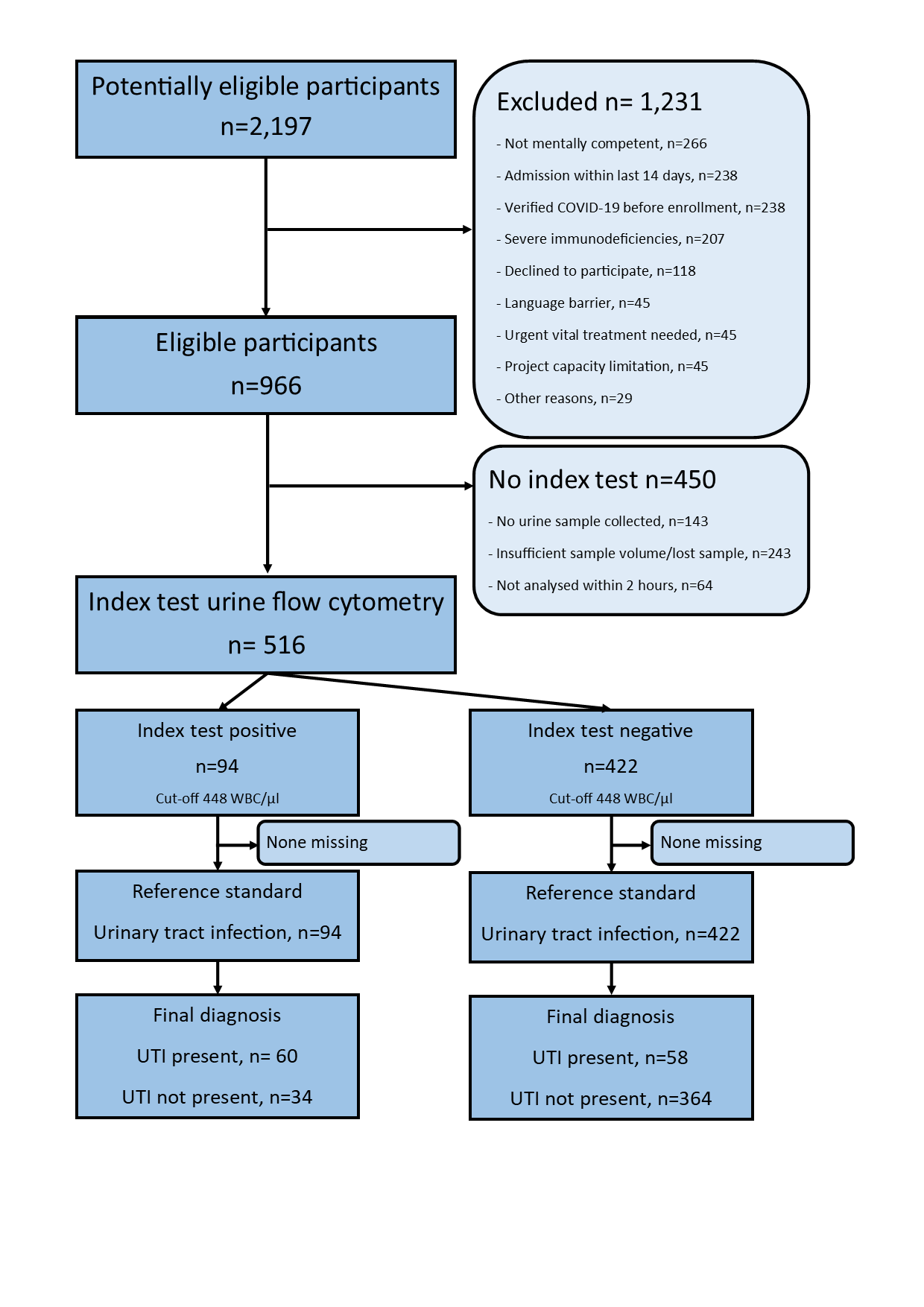

Supplement: Supplementary file 1 [file diagnostics-14-00412-s001.zip › Figure S5 STARD flowchart index test UFC reference test UTI model WBC448.png]

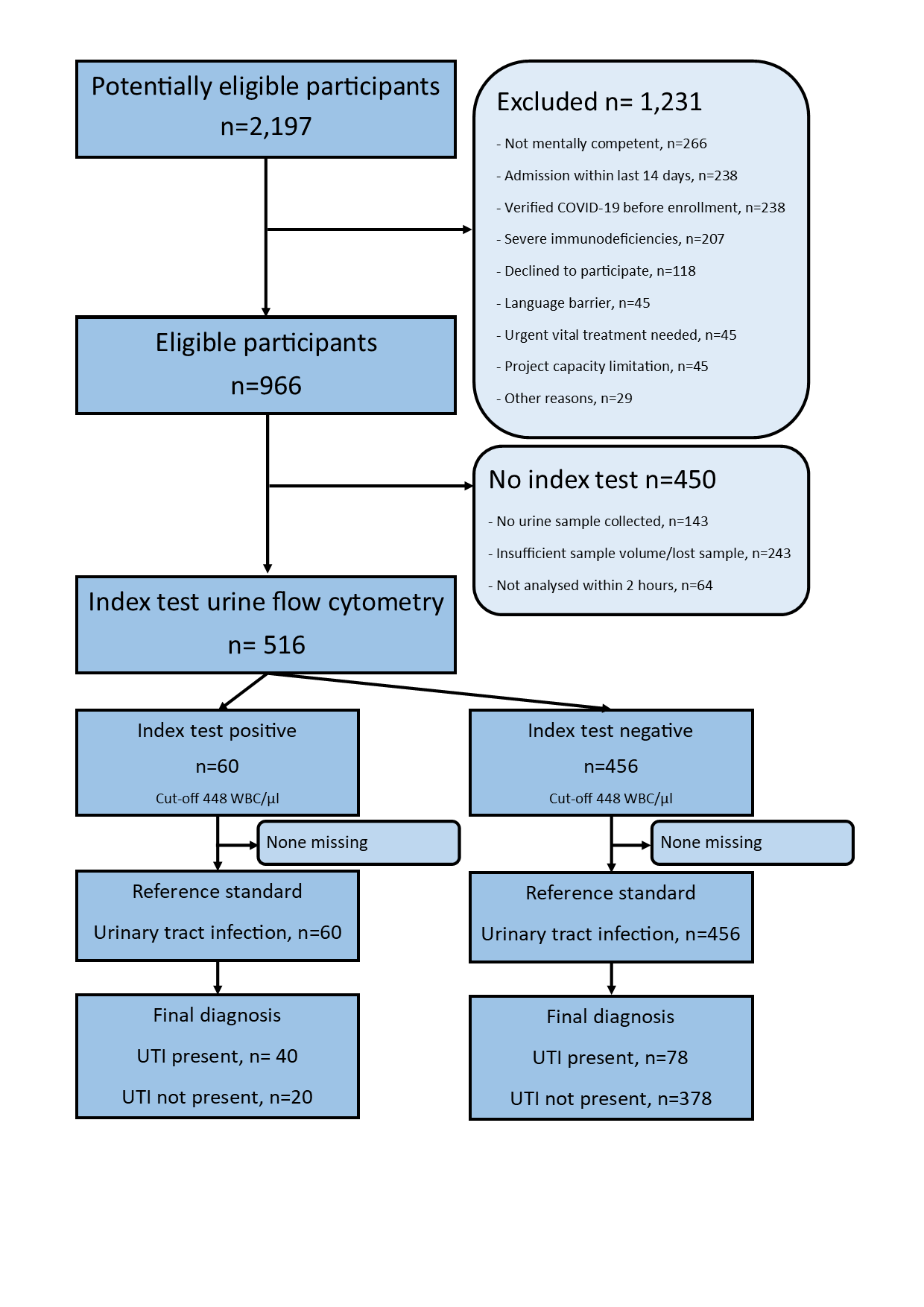

Supplement: Supplementary file 1 [file diagnostics-14-00412-s001.zip › Figure S6 STARD flowchart index test UFC reference test UTI model WBC1125.png]

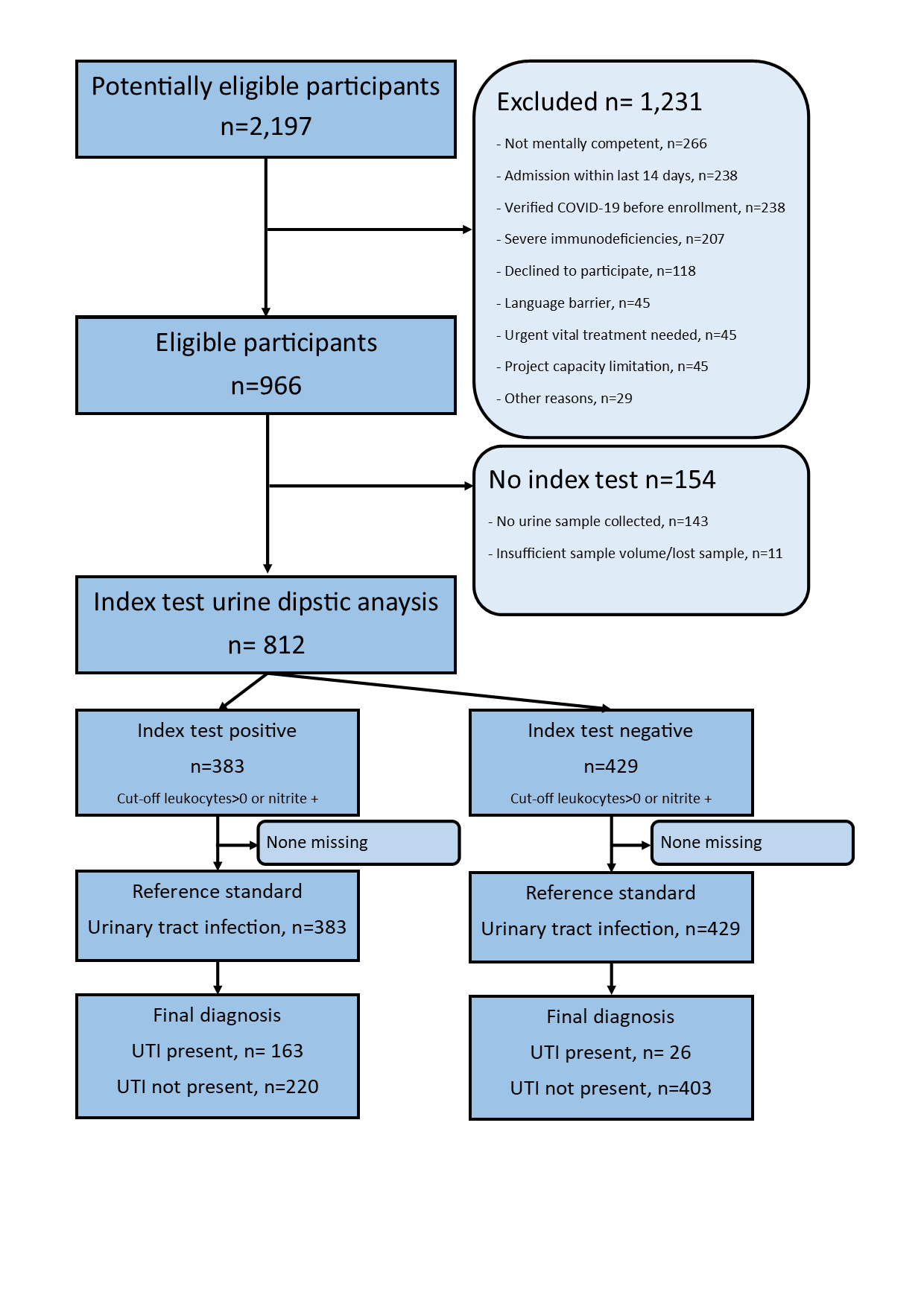

Supplement: Supplementary file 1 [file diagnostics-14-00412-s001.zip › Figure S7 STARD flowchart index test UDA reference test UTI model LKC+ or Nitrite+.png]

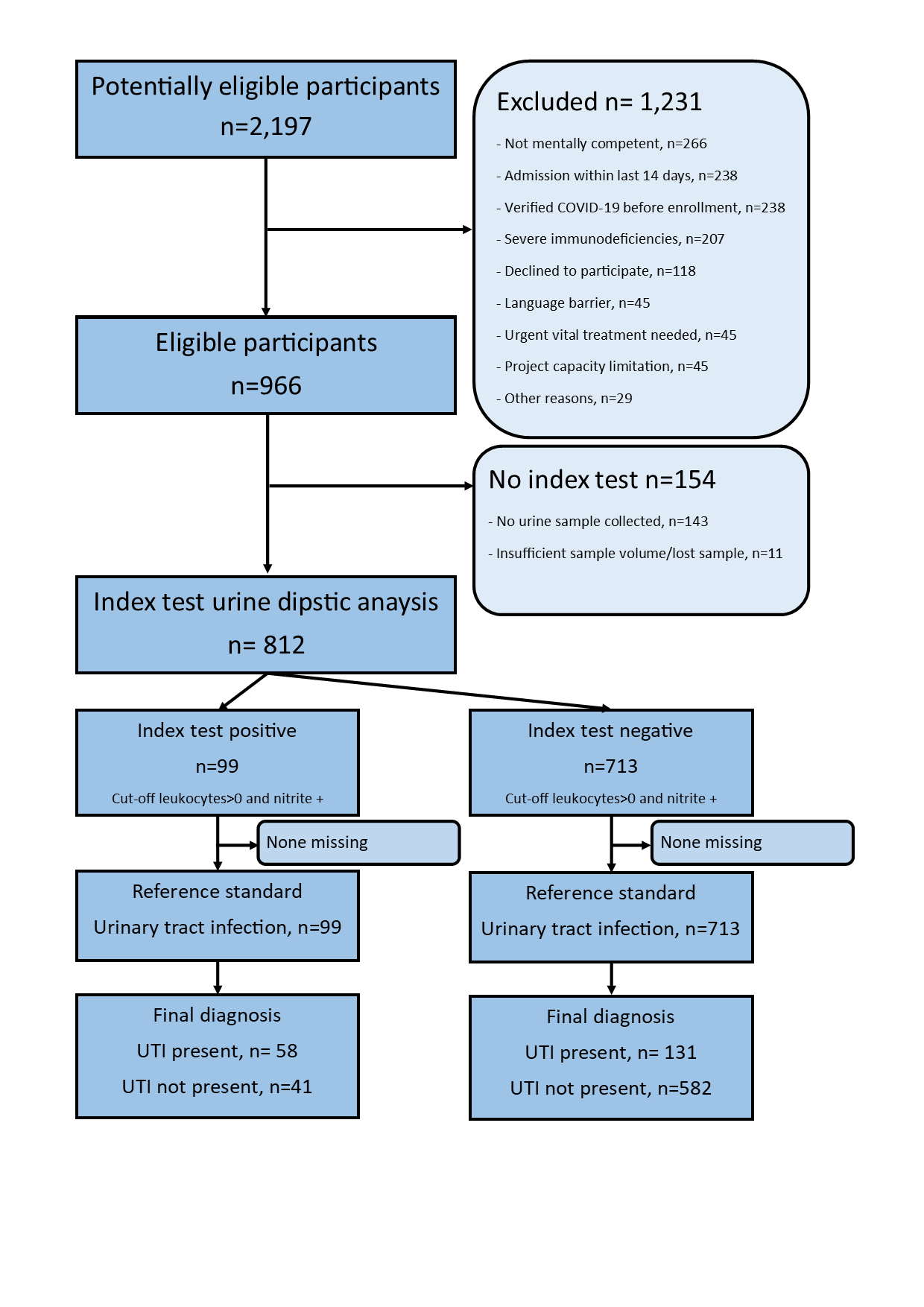

Supplement: Supplementary file 1 [file diagnostics-14-00412-s001.zip › Figure S8 STARD flowchart index test UDA reference test UTI model LKC+ and Nitrite+.png]

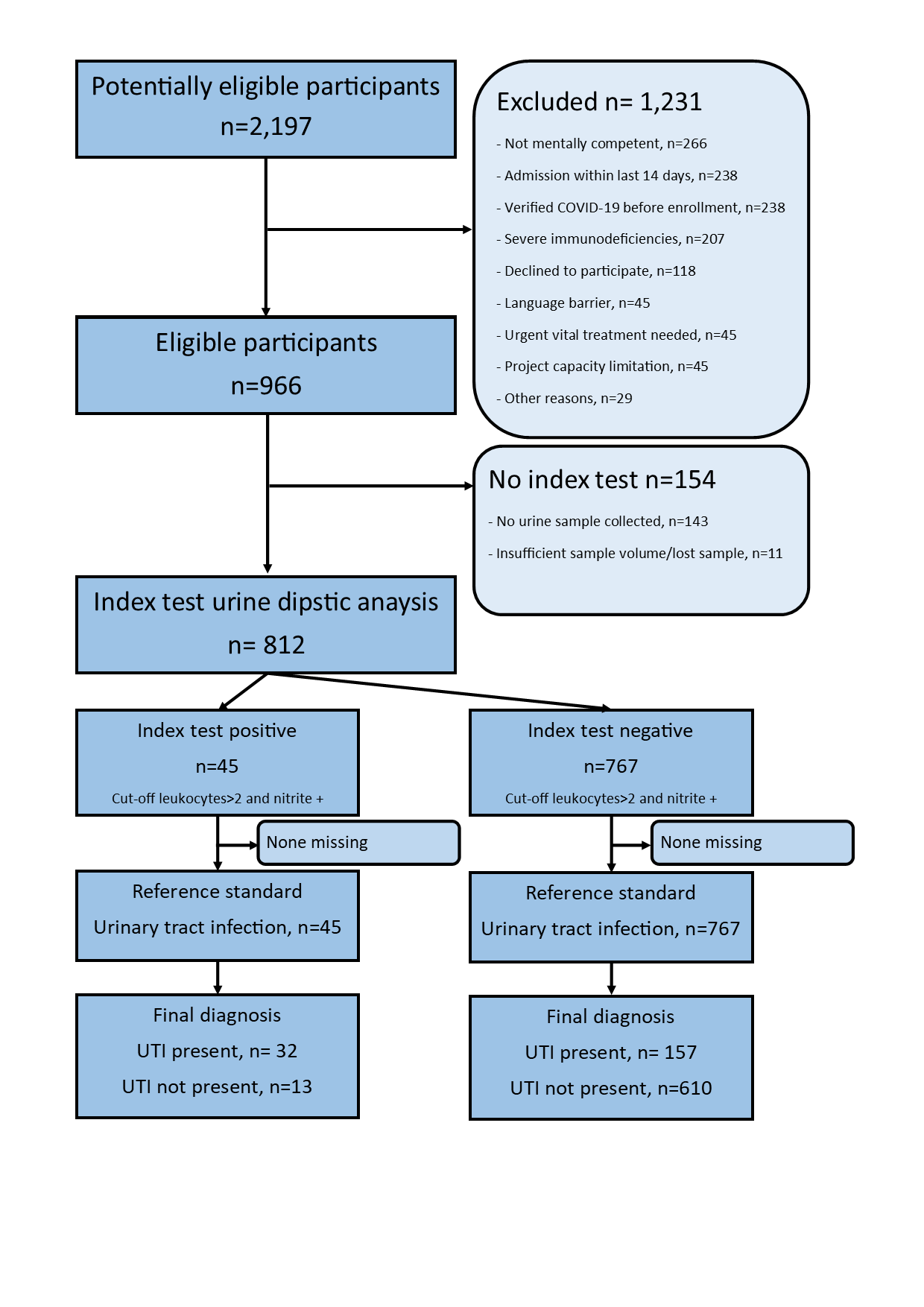

Supplement: Supplementary file 1 [file diagnostics-14-00412-s001.zip › Figure S9 STARD flowchart index test UDA reference test UTI model LKC3+ and Nitrite+.png]
